# Magnitude of common mental disorders and factors associated among people living in Addis Ababa Ethiopia 2018: community based cross-sectional study

**DOI:** 10.1186/s12888-022-03783-9

**Published:** 2022-03-03

**Authors:** Yodit Habtamu, Kalkidan Admasu, Mikiyas Tullu, Alem Kebede

**Affiliations:** 1 Department of Psychiatry, Amanuel Mental Specialized Hospital, Addis Ababa, Ethiopia; 2 Psychosocial and Rehabilitation Services Department, Amanuel Mental Specialized Hospital, Addis Ababa, Ethiopia

**Keywords:** Common mental illness, SRQ-20, Magnitude, Ethiopia

## Abstract

**Background:**

Common mental disorders are a group of distress states manifesting with anxiety, depressive and unexplained somatic symptoms, affecting individuals in different age groups, causes suffering to the individuals, families and community.

**Objective:**

This study assessed the magnitude of Common mental disorder and associated factors among people living in Addis Ababa, Ethiopia.

**Methods:**

Community based cross sectional study design was conducted from November 1 to 30, 2018 among people living in Addis Ababa, Ethiopia. Multistage sampling technique was used to get a total of 755 samples. Common Mental Disorder was assessed through interview using Self-Reported questionnaire (SRQ-20). The collected data were coded, entered into EPI-Info 7 and analysed by using SPPS version 20. Descriptive, analytical statistical procedure; bivariate and multivariate binary logistic regressions with odds ratios and 95% confidence interval was employed. The statistical significance was accepted at *p* value < 0.05.

**Result:**

In this study a total of 723 study subjects were participated, with response rate of 95.7%.The prevalence of common mental disorders was 24.7% with [95%CI; 21.6 – 27.7]. Females (AOR=2.1; 95% CI; 1.39- 3.23), Divorced/widowed (AOR=2.55; 95% CI; 1.16- 5.59), daily labourers (AOR=2.52; 95% CI; 1.3- 4.88, chronic medical illness (AOR=4.5; 95% CI; 2.46- 8.24). are independent predictors of CMD and educational status (primary, secondary and diploma) was positively associated with CMD. in this study. Regarding education (primary, secondary and diploma) (AOR=0.34; 95% CI; 0.17-0.66) and (AOR=0.35; 95% CI; 0.19-0.67) has positively associated with common mental disorders.

**Conclusion:**

The prevalence of common mental disorders was found high. Female sex, marital status like Divorced/Widowed, daily labour workers and chronic medical illness were found to be independent predictors of CMD and educational status (primary, secondary school and diploma holders) was were found to be protective factors.

## Introduction

Common Mental Disorders, those disorders that are characterized by a combination of mild depression, mild anxiety and medically unexplained physical symptoms, seem to affect significant number of people in any community. Such disorders are reported to be highly prevalent and has a huge impact at the individual, family and community levels [1] at large, causing significant burden in a country’s healthcare system [2, 3]*.*

It is repeatedly articulated that no one is immune from mental illness. This means anyone in every corner of the globe is vulnerable to any form of mental health problems in their life time. This fact led to the recognition that mental health conditions truly have public importance [4, 5]*.* According to a report from WHO, 25% of the world population will develop at least one type of mental illness in their life time [6]*.* Due to the high rate of relapse and related complications, almost one out of the ten global burden of diseases is represented by CMD [7, 8]. According to WHO, unipolar disorder is the fourth leading cause of morbidity and premature death. Additionally, depression, on top of being the most common disorder contributing to suicide, it is also associated with high levels of morbidity and mortality [7, 9]. And with approximately 6% of total disability, it is projected that depression will be the leading cause of disease burden globally by the year 2030 [10].

In developing countries like Ethiopia, there are many barriers which affect mental health care [11]*.* Ethiopia’s health service delivery arrangement is structured into three tiers providing primary, secondary and tertiary level healthcare. However, contrary to its national health policy which prioritizes care delivery at primary health-care units, which intern are composed of various health posts, health centres and primary hospitals, there is a huge treatment gap among CMD patients who visit these settings. Mental health problems such as CMDs are not well-recognized within these settings. Help-seeking for such disorders is most often limited to the family or local community, and as a result, those who are living with these disorders usually remain undetected in the health system. This leads to ineffective treatment options that usually creates a missed opportunity for suicide prevention.

Studies in the field of mental health report various findings with respect to specific mental health issues in general and CMDs in particular. For example, surveys from Australia and the US community reported a twelve-month prevalence estimates of mood and anxiety disorders to range from 6.6% to 18.1% [12].. In South American countries like Brazil, Santiago and Chile, the prevalence of CMDs was reported to be 29.9% and 25% respectively [1, 13]. In Britain, the estimated prevalence of CMDs is reported to be 24.6% [14] and yet another finding in Britain with different study setting reported a prevalence estimate of 24.2% [15]. In the European countries of Greece and Sweden, the magnitude of CMDs was reported to be high, ranging from 14-17.2 %, while in eastern Asia, the prevalence was reported to be around 8.8 % [16–18].

In Africa, various studies revealed different CMD prevalence results in population-based surveys. In Kenya and South Africa for example, the prevalence of CMDs was reported to be 10.8% and 34.9% respectively. In another study conducted in Nigeria, the life time prevalence of CMDs was found to be 12.1% and a twelve-month prevalence was reported to be 5.8% [19–21]*.*

In Ethiopia, the various community-based studies conducted in different parts of the country reported that the prevalence of CMDs range from 11.2% to 33.4% [22–28].

Concerning the factors associated with common mental disorders, female sex, low educational status, older age, unemployment, family history of mental illness, chronic physical health problems and substance use were found to be the strong predicators of CMDs in different literatures [23, 29–31].

This study is designed to assess the prevalence and the corresponding associated factors for CMDs among people living in Addis Ababa. Addis is Ethiopia’s capital city where most people from different corners of the country migrate in from time to time for better job, education and training opportunities. Although such influx from rural areas into such urban settings is a common feature, it also has its cost in terms of the long term mental health effects of such inhabitants [32]. Various studies [33] have reported the correlation between increased urbanization and its deleterious consequences on mental health attributing factors such as the influence of increased stressors; adjustment difficulties; adverse life events such as overcrowding, polluted environments, poverty and dependence on cash economy; high levels of violence and reduced social support for the stated impacts.

Thus, this study will provide significant evidence on the magnitude of the problem in the city emphasizing on the need for appropriate and timely intervention with respect to the integration of the country’s mental health service provision across its primary health care units for the purpose of managing various mental health conditions at large and CMDs in particular.

## Objectives

The aim of this study was to assess the magnitude of CMDs and associated factors among people living in Addis Ababa, Ethiopia.

## Materials and methods

### Study setting and period

This study was conducted from November 1 to November 30, 2018 in Addis Ababa, Ethiopia. Addis Ababa is the capital city of Ethiopia with an estimated population of 2,738,248 inhabitants [34] the country’s third population and housing censes report, of which 52.36% are women. It has an annual population growth rate of 2.1%, and an estimated population density of 5165.1 people per square kilo meter. Addis has an elevation of 2,355 meters above sea level and is located in the geographic centre of the country being surrounded by chains of hills and mountains. Administratively, the city is structured in 10 sub cities and 99 woredas.

Addis Ababa was selected as the study area for various reasons. First and for most, Ethiopia, as a country, is undergoing a fast economic growth that is also changing its urban landscape. Its rural suburban areas have found themselves in the middle of cities and towns and conurbation is taking place in some of its urban centres, making Addis a metropolis that hosts increasing number of migrants each year [34]. As the city is the political, economic and social hub of the country, almost one out of five of its dwellers live there while many people migrate from their hometown to the city every year, of which 57% account for rural migrants [35]. All of the ethnic groups in the country are represented in the city, with the Amhara, Gurage, Oromo, Tigre, Somali and SNNP (Southern Nations, Nationalities and Peoples) account for 90% of its inhabitants [34].

### Study design, population and sampling

A community-based cross-sectional study was conducted and multistage sampling technique was used to select 755 participants. Initially, 3 sub-cities were selected from the 10 administrative sub-cities using simple random sampling technique. The selected 3 sub-cities are comprised of 10 woredas each, making a sample pool of 30 woredas, and from these 30 woredas, 8 Woredas were again selected using the same simple random sampling technique. In order to ensure representativeness of the sample, the principle of proportional allocation was followed in the selection of the woredas located in each sub-city. Then, the same lottery method was again used to select possible households from each woredas. The main breadwinner of the household, be it a man or a woman, was taken as a representative of the household and thus was taken as the study unit. During the interview process, when the selected study unit becomes unavailable, the interviewers had to revisit the household on the following day and other three consecutive days, and if the subject was still unavailable during those visits, the next in-line household was considered for the interview. The lottery method was again used to select between in-line households if they happen to be more than one. The study included all adults residing in Addis Ababa who were >18 years of age who also were available at the time of data collection. Despite their availability, however, Subjects who were seriously ill and who were unable to communicate were excluded from the study.

### Sample size determination

The minimum number of sample size required for this study was determined using single population proportion formula considering the following assumptions : [36]

$$\mathrm{n}=\frac{{\left(\mathrm{Z}\upalpha /2\right)}^2\mathrm{p}\ \left(\mathrm{l}\hbox{-} \mathrm{p}\right)}{{\mathrm{d}}^2}$$

*Where*

n = minimum sample size required for the study

Z= Standard normal distribution with confidence interval of 95%, Z=1.96

P= Proportion of the prevalence of CMDs conducted in Jimma town in 2015 which was (33.6%)

[28], ; hence, P= 33.6 % (0.336) was used

d= Absolute precision or tolerable margin of error (d) =5%=0.05

g=design effect (D=2) was used, because of multistage sampling technique : [37]

$$\mathrm{n}=\frac{\mathrm{g}{\left(\mathrm{Z}\upalpha /2\right)}^2\mathrm{p}\left(\mathrm{l}=\mathrm{p}\right)}{{\mathrm{d}}^2}=\frac{(2)\mathrm{x}(1.96)2\times 0.336\left(1\hbox{-} 0.336\right)}{(0.05)^2}=\underset{=}{686}\kern0.75em$$

Then adding 10% (686 x 0.1 = 68.6 ≈ 69) of non-respondents, the total sample size for this study was 686+69 = 755*.*

### Data collection technique

To ensure the quality of data, the SRQ items were adopted accordingly. The items were initially translated into Amaharic language and then translated back to the English language. Reliability test was conducted and the yielded internal consistency score was 0.84 [38]. Pre-test was done one week prior to the actual data collection on a population of 5% of the determined sample size who were residents of other sub-cities that were not included in the main survey. Data collectors were trained by the principal investigators for 5 consecutive days on the topics of Introduction to Common Mental Disorders, Research Methods, Interviewing Skills, Sampling, Participant Recruitment, Consent Solicitation and Research Ethics. Data were collected by sixteen nurses who hold a BSc degree in Clinical Nursing and work as health extension workers at various primary health care units in the city. They are recruited as data collectors on an electronic advertisement posted on social media. During the data collection process, they were regularly supervised by four senior psychiatry professionals who hold a Master’s degree in ICCMH (Integrated Clinical and Community Mental Health) and has a significant research background. These supervisors were referred for this project on a snowball basis. The collected data were reviewed and checked for completeness, consistency and relevance jointly by these supervisors and one of the assigned principal investigators for that day.

### Study variables and measures

#### Variables:

##### Dependent variable

Common Mental Disorder (Yes/ No) : CMD was measured using 20 items SRQ using a cut of point of > 7. Subjects who score less than 7 were considered to have no CMD and those who score greater than or equal to7 were considered as having a CMD.

##### Independent variables include

Socio-demographic Factors **(**Age, Sex, Marital Status, Religion, Educational Status, Occupation, Gross Family Monthly Income**)** ; Clinical Factors **(**History of Chronic Medical Illness**,** Previous History of Mental Illness and Family History of Mental Illness) and Current Substance Use.

#### Operational definitions

##### Primary healthcare units (PHCU)

These are health care settings comprised of health posts, health centres and primary hospitals. Health Posts provide various preventive and health promotion services for a rural population load of 3000-5000 people. Health Centres provide both preventive and curative services for both semi-urban and urban population load of 15,000-25,000 people. They also serve as referral centres and practical training sites for HEWs. Primary Hospitals offer various inpatient and ambulatory services for a population of 60,000-100,000 people. They also provide emergency surgery services (including caesarean sections and blood transfusions)

##### Health extension workers (HEWs)

are professional clinical nurses who work at the primary healthcare units (the health posts in the context of rural areas and the health centres in the context of urban areas). They are assigned to directly work in the community by making house to house visits and are responsible for treating cases such as malaria, pneumonia, scabies, trachoma, and other mild illnesses. They also give health education, first aids and client referrals to health centres for services requiring higher-level care.

### Measurements

Data was collected in a face-to-face interview using structured questionnaires. Data on magnitude of CMDs was collected using Self-Reported Questionnaire (SRQ-20) which was developed by the WHO. It was used to assess the presence and also the prevalence of CMDs and neurotic symptoms of anxiety, depression, and medically unexplained somatic complains. Though the SRQ was originally designed as a self-administered scale, it also was reported to be suitable for being administered by an interviewer due to the low literacy rate in developing countries [19]*.* It is scored using nominal scales of 0 or 1. A score of 1 indicates the presence of symptoms in the past one month and a score of 0 indicates no symptoms at all. The total score is obtained by summing the individual scores from each 20 items. Higher score indicates the probable existence of CMD, while lower score indicates non-existence of CMD. A cut of point score of > 7 was used in this study [22]*.* The SRQ had previously been translated into Amharic and validated in Ethiopia, and it has been used for community surveys [39].

Data on Socio-demographic factors (Age, Sex, Marital Status, Religion, Educational Status, Occupation, Gross Family Monthly Income) was collected using techniques derived from different literatures. The Clinical Variables are composed of three entities; History of Chronic Medical Illness such as (Tuberculosis, HIV/AIDS, Diabetics**,** Cardiovascular diseases, Asthma, Hypertension and Cancer) , Family History of Mental Illness and assessment of Current Substance Use that assesses usage of addictive substances of khat, alcohol, tobacco, and cannabis within the last three months.

### Data processing and analysis

Initially, the data was checked for completeness, relevance and consistency. It then was coded and entered into the computer using Epi Info version 3.5.1 and analysed using SPSS version 20 softwares. Descriptive statistic was used to explain the study participants in relation to the study variables. Binary logistic regression was fitted to the data and Hosmer-Lemeshow goodness with the (Chi- square 11.76 and *p*-value 0.157) and maximum likelihood were checked and *P* value less than 0.05 was considered as a statistically significant point. Bivariate and Multivariate binary logistic regression was conducted to determine the presence of statistically significant association between the explanatory variables and the outcome variables. Variables with *P*-value less than 0.2 during the bivariate logistic regression were selected for further analysis in the Multivariate logistic regression. The strength of the association was presented by odds ratio with 95% C.I. Multicolliniarity was tested using tolerance and variance inflation factors.

### Ethical approval and consent to participate

Ethical Clearance was obtained from Amanuel Mental Specialized Hospital’s ethical review committee. A formal letter of permission was obtained from the hospital. Written informed consent was obtained from each study participants after explaining the purpose and benefit of the study. This study was conducted in accordance with the Declaration of Helsinki. Which states that participation is voluntary, and that clients have the right to withdraw from completing the questionnaires at any given time they wish without giving a reason. Participants were also priorly informed that there were no associated expectations, additional treatments, associated benefits and/or risks of any kind to be rendered or acquired as a result of their participation in the study. Those participants who were found to have CMDs during the study were further assessed by the ICCMH Supervisors for their conditions and were then linked to a nearby Health Centre’s Psychiatric Clinic for a more comprehensive treatment and follow-up options.

## Result

### Socio- demographic characteristics

A total of 723 study subjects participated in this study, making a response rate of 95.7%. More than half of the participants (56.7%) were females; half of them (50.8%) were married ; more than one-third of them (35.4%) were from the Amhara ethnic group ; more than half of them (63.2%) belong to the Orthodox Christian religion ; nearly one-third of them (28.5%) completed a secondary school education ; more than one-third of them (37.3%) were within the age group of 25-34 ; more than one-fourth of them (25.9%) work as daily labourers and more than one-third of the participants (40.8%) earn a monthly income ranging from 100-800 ETB. (Table 1).

**Table 1:** Distribution of socio demographic and economic characteristics of study participants in Addis Ababa, Ethiopia, 2018 (*n*=723)

Variables		Frequency	Percentage
Age	18-24	75	10.4
	25-34	270	37.3
	35-44	190	26.3
	>45	188	26.0
Sex	Male	313	43.3
	Female	410	56.7
Marital status	Married	367	50.8
	Single	297	41.1
	Others^a^	59	8.2
Religion	Orthodox	457	63.2
	Protestant	116	16.0
	Muslim	128	17.7
	Others^b^	22	3.0
Ethnicity	Amahara	256	35.4
	Oromo	186	25.7
	Tigre	74	10.2
	Gurage	147	20.3
	Others^c^	60	8.3
Educational status	Unable to read and write	80	11.1
	Primary school	131	18.1
	High school	206	28.5
	Diploma	187	25.9
	Degree and above	119	16.5
Occupational status	Private employed	178	24.6
	Government employed	172	23.8
	Daily laborer	187	25.9
	Unemployed	98	13.6
	Others^d^	88	12.2
Gross family monthly	100-800	295	40.8
	801-1500	127	17.6
Income(ETB)	1501-3000	156	21.6
	>3000	145	20.1

Note ^a^Divorce, Widowed and Separated. ^b^ Catholic and Jehovah witness. ^c^ Hadiya and Welayita. ^d^ Pensioners and house servant

### Clinical variables and substance use of respondents

Of the total participants, 170 (16.2%) had a history chronic medical illness while 94 (15.1%) had family history of mental illness. (Table 2). Regarding substance use, from the total study participants, 160 (22.1%) reported history of drinking alcohol within the last three months whereas 34 (4.7%) were using tobacco products. 54 of them (7.5%) used khat and 16 (2.2%) used cannabis.

**Table 2:** Distribution of clinical factors of study participants in Addis Ababa, Ethiopia, 2018 (*n*=723)

Variables		Frequency	Percentage
Previous history of mental illness	Yes	117	16.2
	No	606	83.8
Family history of mental illness	Yes	94	13.0
	No	629	87

### Prevalence of common mental disorders and distribution of symptoms

**Fig 1:**
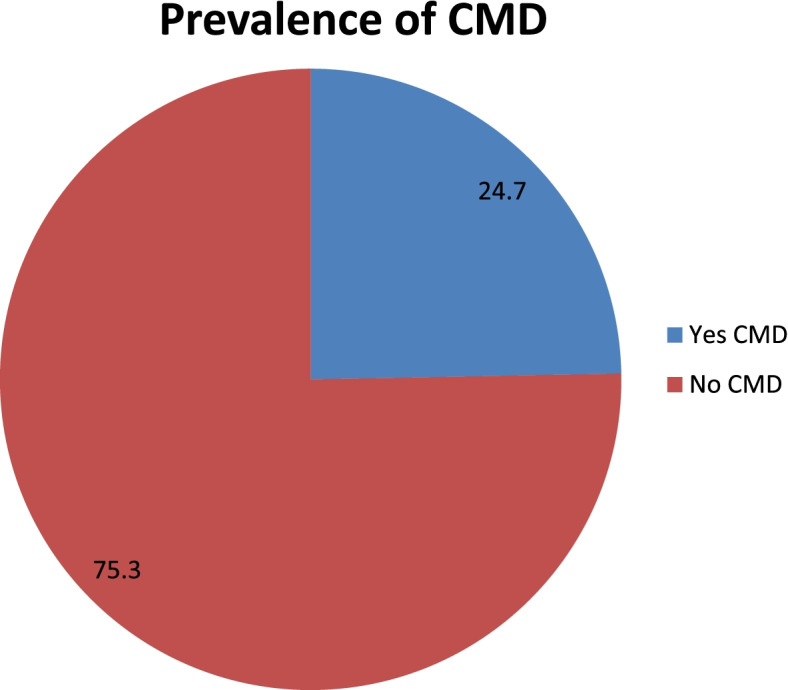
Prevalence of Common Mental Disorder among living in Addis Ababa, Ethiopia, 2018

The overall prevalence of CMDs in this study was found to be 178 (24.7%), with [95% CI; 21.6– 27.7] Among the participants, 62.9% of them were females and 37.1% of them were males. (Fig 1). The majority of the study participants reported that out of the total twenty symptoms listed in the SRQ, headache, being easily tired, Sleeping badly, feeling nervousness, tightness or worried and Feeling tired all the time during the last 30 days were the most commonly reported symptoms (Table 3).

**Table 3:** Distribution of SRQ symptoms among the study participants (*n*=723) living in Addis Ababa, Ethiopia, 2018

Symptomes	Frequency	Percent
Headache	352	48.7
Easily tired	219	30.3
Sleep badly	217	30
*Feel nervous, tens or worried*	197	27.2
*Feel tired all the time*	185	25.6
*Poor appetite*	185	25.6
*Lost interest in things*	184	25.4
Difficult enjoy daily activity	155	21.4
Thought of ending life	155	21.4
Poor digestion	152	21
Difficult in decision making in day to day activity	149	20.6
Feel unhappy	140	19
Daily works suffering	132	18.3
Uncomfortable feeling in the stomach	114	15.8
*Feel worthless person*	105	14.5
*Unable to play a useful part in life*	102	14.1
Others	262	36.3

### Factors associated with common mental disorders

Multivariate logistic regression was carried out in relation to a number of variables which could likely be expected to influence CMDs (Table 4).

**Table 4:** Bivariate and Multivariate analysis of factors associated with common mental disorders among respondents living in Addis Ababa, Ethiopia, 2018

Variables	Total (f)	CMD		COR(95% CI)	AOR(95% CI)
		No	Yes		
Sex					
Female	410(56.7%)	298 (54.7%)	112(62.9%)	1.41(0.99-1.99)	2.1(1.39-3.23)***
Male	313(43.3%)	247(45.3%)	66(37.1%)	1	1
Age					
18-24	75(10.4%)	54(9.9%)	21(11.8%)	1	1
25-34	270(37.3%)	229(42%)	41(23%)	0.46(0.25-084)	0.56(0.28-1.12)
35-44	190(26.3%)	142(26.1%)	48(27%)	0.87(0.48-1.58)	1.19(0.57-2.46)
>45	188(26%)	120(22%)	68(38.2%)	1.45(0.81-2.61)	2.03(0.92-4.48)
Marital status					
Single	297(41.1%)	227(41.7%)	70(39.3%)	**1**	1
Married	367(50.8%)	286(52.5%)	81(45.5%)	0.92(0.64-1.32)	1.12(0.66-1.88)
Divorced/widowed	59(8.2%)	32(5.9%)	27(15.2%)	2.7(1.54-4.88)**	2.55(1.16-5.59)*
Educational status					
Unable to read and write	80(11.1%)	48(8.8%)	32(18%)	1.67(0.92-3.03)	0.49(0.2-1.21)
Primary school	131(18.1%)	89(16.3%)	42(23.6%)	1.18(0.69-2.03)	0.39(0.18-0.83)*
High school	206(28.5%)	164(30.1%)	42(23.6%)	0.64(0.38-1.08)	0.34(0.17-0.66)**
Diploma	187(25.9%)	159(29.2%)	28(15.7%)	0.44(0.25-0.78)**	0.35(0.19-0.67)**
Degree and above	119(16.5%)	85(15.6%)	34(19.1%)	**1**	**1**
Occupational status					
Government employed	172(23.8)	145(26%)	27(15.2%)	1	1
Private employed	178(24.6%)	135(24.8%)	43(24.2%)	1.71(1.0-2.92)*	1.76(0.92-3.35)
Daily labourer	187(25.9%)	127(23.3%)	60(33.7%)	2.54(1.52-4.24)***	2.52(1.3-4.88)**
Unemployed	98(13.6%))	74(13.6%)	24(13.5%)	1.74(0.94-3.23)	1.35(0.6-3.01**)**
Others****	88(12.2%)	64(11.7%)	24(13.5%)	2.01(1.07-3.76)*	0.45(0.24-0.83)
Gross family monthly Income(ETB)				1	1
100-800	295(40.8%)	202(37.1%)	93(52.2%)	1	1
801-1500	127(17.6%)	103(18.9%)	24(13.5%)	0.51(0.31-0.84)*	0.84(0.46-1.55)
1501-3000	156(21.6%)	119(21.8%)	37(20.8%)	0.67(0.43-1.05)	1.31(0.72-2.39)
>3000	145(20.1%)	121(22.2%)	24(13.5%)	0.43(0.26-0.71)**	0.58(0.29-1.16)
Chronic medical illness					
Yes	117(16.2%)	69(12.7%)	48(27%)	2.55(1.68-3.86)***	4.5(2.46-8.24)***
No	606(83.8%)	476(87.3%)	130(73%)	1	1
Family hx of Mental illness					
Yes	94(13%)	78(14.3%)	16(9%)	0.59(0.33-1.04)	0.45(0.24-0.83)
No	629(87%)	467(85.7%)	162(91%)	1	1

Females were 2.1 times more likely to develop CMDs than males (AOR=2.1; 95% CI; 1.39- 3.23). Those who are Divorced/Widowed were 2.55 times more likely to develop CMDs than the singles (AOR=2.55; 95% CI; 1.16- 5.59). Working as a daily labourer as a form of ones occupation increases the chance of developing CMDs by 2.52 times than working on government jobs. (AOR=2.52; 95% CI; 1.3- 4.88). It was also found that those who have a prior history of chronic medical illness were 4.5 times more likely to develop CMDs than those who have no history of medical illness at all (AOR=4.5; 95% CI; 2.46- 8.24). Regarding Educational Status, the odds of developing CMDs among those who completed their primary education, secondary education and diploma-level tertiary education were decreased by 61%, 66% and 65% when compared to those degree holders (AOR=0.39 ; 95% CI; 0.18-0.83), (AOR=0.34; 95% CI; 0.17-0.66) and (AOR=0.35 ; 95% CI; 0.19-0.67) respectively.

## Discussion

The finding from the current study showed that the estimated prevalence of CMDs was 24.7%. This finding was in line with studies conducted in Britain at different study settings that reported a prevalence estimate of 24.6% and 24.2% respectively [14, 15]. It was also in line with another study conducted in Ethiopia’s Illubabore town which reported a prevalence rate of 27.2% [27]. The possible reasons for the similarity might be attributed to the nature of the instrument (SRQ-20) used in all the studies.

Another study conducted in Kenya reported a prevalence rate of 10.8% [19] while a study conducted in Nigeria reported a prevalence rate of 5.8% [21]. The possible reason for the difference in the reported magnitudes might be attributed to the tool (CIS-R, CIDI) used in the studies.

This study was found to be lower from a study conducted in South Africa, which reported a magnitude of (34.9%) [20]. The possible reasons for such differences in the reported estimates might actually be attributed to the tool used in the assessment (the CIS-R, CIDI).

Studies conducted in the European countries of Greece and Sweden reported a prevalence rate ranging from 14-17.2% while another study conducted in Eastern Asia reported a magnitude of 8.8%. The difference in the reported outcomes might be attributed to the cultural differences in the countries and the socioeconomic status of the populations the studies address.

Other similar studies conducted in the Ethiopian towns of Kombolcha reported a rate of 32.4% while a study in Jimma reported a magnitude of 33.4 % [26, 28]. The deference might be due to the different cut-off point used for the tool (SRQ-20) (The Kombolicha study used a cut-off point of >=8). The other possible reason might be a population difference.

This study however reported a rather higher magnitude when compared with other studies previously conducted in the different parts of the country. For example, a study conducted in Butajira town reported a prevalence estimate of 17% ; a similar study conducted in Harar city reported a prevalence estimate of 14.9% ; another study conducted in Hadiya town reported a prevalence estimate of 11.2% and yet another study conducted in Addis Ababa reported a rate of 11.7%. The difference might be attributed to the increased urbanization and conurbation effect the city witnessed through time. The previous studies were conducted in the past two decades, and since then, there might have been a considerable change in the population dynamics and associated concomitant factors with a potential to affect the mental health of the inhabitants’ in particular and the community at large.

This study however reported a rather higher magnitude when compared with other studies previously conducted in the different parts of the country. For example, a study conducted in Butajira town reported a prevalence estimate of 17% ; a similar study conducted in Harar city reported a prevalence estimate of 14.9% ; another study conducted in Hadiya town reported a prevalence estimate of 11.2% and yet another study conducted in Addis Ababa reported a rate of 11.7%. The difference might be attributed to the increased urbanization and conurbation effect the city witnessed through time. The previous studies were conducted in the past two decades, and since then, there might have been a considerable change in the population dynamics and associated concomitant factors with a potential to affect the mental health of the inhabitants’ in particular and the community at large.

Being Female, being widowed/divorced, working as a daily labour and having a chronic medical illness were found to be the independent predictors of CMDs while educational status (completing primary, secondary and diploma-level tertiary education) was found to have a positive association with CMDs.

Females were 2.1 times more likely to develop CMDs than males. This finding is also supported by studies conducted in Greece, South Africa and even Ethiopia [17, 20, 23, 27]. The possible reason might have emanated from biological cause of hormonal difference [40]. The other articulated reason is a social cause where poverty, vulnerability and disadvantaged background might have played a role. Due to their social structure and low socio-economic status, the burden of social, familial and household responsibly usually fall on women. They usually are disadvantaged of schooling and are also highly vulnerable for various physical, emotional and sexual abuses. These cumulated factors might have predisposed them to a higher risk for CMDs.

The Divorced/widowed were 2.55 times more likely to develop CMDs than the single. This finding is supported by other studies conducted in Santiago and Chilly [13]. The possible reason could be the extra household responsibilities assumed by these individuals following a change in their social status that might have imposed a financial, emotional and physical strains.

Daily labourers were 2.52 times more likely to develop CMDs than government employees. The possible reason could be their low wage scale, insecurity at their jobs, and occupational safety. Such factors can lead to stressful and unsafe situations that might trigger CMDs. Different theories state that low income can be explained in part due to poverty. People who experience poverty face significant challenges to fulfil their basic needs. It interferes with their ability to participate in productive activities, which in turn hampers their ability to build and maintain sustaining social relationships, and ultimately creating a significant stigma and discrimination.

With respect to educational status, the odds of developing CMDs among those who completed their primary, secondary and diploma-level tertiary education was decreased by 61%, 66% and 65% respectively when compared to those who completed a degree-level education. The possible reason could be explained by the fact that having higher education increases access to the most professional jobs and enhance social capital, ultimately decreasing the feelings of insecurity and vulnerability [41].

Those who suffer from a chronic medical illness were 4.5 times more likely to develop CMDs than those with no history of chronic medical illness. This finding is in line with studies conducted in Kenya, Taiwan and Brazil [1, 16, 19]. The possible reason could be those who are living with chronic illness might have limited daily activities as their illness might interfere with their daily functionality and as a result, might experience dissatisfaction in life, which might as well expose them for emotional strains, and ultimately to depression and anxiety.

### Strength and limitations of the study

The study was conducted in a short period of time. Nonetheless, the consistently stable reliability and construct validity of the SRQ scale attained in the current study can be used as reference in other subsequent studies.

However, the research design, the study being a cross sectional study, has prevented us from asserting possible causal relationships for the reported associations. In addition, social desirability bias might also have occurred among the study subjects during interviewing. At last, as the results are drawn from probabilistic samples, they can only be generalizable to populations with similar underlying profiles, that is, the context of a low/middle income countries, and not to the high-income countries.

## Conclusion and recommendations

In this study, the prevalence of CMDs was found to be high. Being Female, Being Divorced/Widowed, Working as a Daily Labourer and Living with a Chronic Medical Illness were found to be the independent predictors of CMDs while Educational status (completing a primary, secondary and diploma-level tertiary education) was found to be a protective factor.

The authors recommend a longitudinal and follow-up studies to be conducted in order to overcome the foreseen limitations that emanate from the current design. As part of a policy recommendations, the authors would like to recommend a strategic intervention that focused on addressing mental health issues. The country’s health policy has a package called urban health extension package and the authors would like to recommend a mental health section to be included in that package. Furthermore, continuous and pervasive training should be facilitated for the Health Extension Workers (HEWs) on proper mental health-focused screening tasks, and it is highly recommended that such screening attempts be supported by evidence-based assessment and screening tools which will help them in properly identifying possible CMD cases during their house to house visits. The authors would also like to recommend that possible referral and linkage systems be established along the country’s three-tier health systems that will easily pave a way for early identification and proper management of identified cases among the community at large and with those special vulnerable groups in particular. Further studies are also recommend for continuous evidence-based interventions.

## Data Availability

All the data included in this manuscript are represented in the form of tables and figures. The non-identifying raw data generated and/or analysed during the current study are not publicly available due to confidentiality issue. They can however be available from the corresponding author on reasonable request through the following email address: yodita25yididya@yahoo.com.

## Abbreviations

CMD: Common Mental Disorders
WHO: World Health Organization
SRQ-20: Self-Reported questionnaire 20
CISD: Critical Incident Stress Debriefing
AOR: Adjusted Odd Ratio
COR: Cumulative Odd Ratio
CI: Confidence Interval
SPSS-20: Stastical Package for the Social Sciences-20
AMSH: Amanuel Mental Specialized Hospital
ICCMH: Integrated Clinical and Community Mental Health Specialists
HEWs: Health Extension Workers
PHCU: Primary Healthcare Units
ETB: Ethiopian Birr
ERC: Ethical Review Committee
